# Early Stages of *we/we wal/wal* Mouse Hair Morphogenesis: Light and Fluorescent Microscopy of the Whole-Mount Epidermis

**DOI:** 10.1155/2014/856978

**Published:** 2014-06-03

**Authors:** Alexandra Rippa, Olga Leonova, Vladimir Popenko, Andrey Vasiliev, Vasily Terskikh, Ekaterina Vorotelyak

**Affiliations:** ^1^Department of Biomedical Technologies, Pirogov Russian National Research Medical University, 1 Ostrovityanova, Moscow 117997, Russia; ^2^Laboratory of Cell Basics for Cancer Research, Engelhardt Institute of Molecular Biology, 32 Vavilov Street, Moscow 119991, Russia; ^3^Laboratory of Cell Proliferation, N.K. Koltsov Institute of Developmental Biology, 26 Vavilov Street, Moscow 119334, Russia; ^4^Department of Cell Biology and Histology, Lomonosov Moscow State University, 1 Leninskiye Gory, Moscow 119234, Russia

## Abstract

In adult skin, hair follicles cyclically self-renew in a manner that recapitulates embryonic hair follicle morphogenesis. The most common pathology of hair in adults is alopecia, which is hair loss to different extent. There are a number of murine models of alopecia including spontaneous mutations. In the present study, we worked with double homozygous *we/we wal/wal* mice which demonstrate symptoms closely resembling human alopecia. Using whole-mount preparations of epidermis of E18.5 embryos we show that hair follicle defects can be revealed as early as during embryonic morphogenesis in these mutants. The number of hair follicles was reduced almost 1.5-fold in mutant skin. The shape of the early stage small follicles was altered in mutant animals as compared to control ones. Additionally, follicles of mutant embryos were wider at the point of conjunction with interfollicular epidermis. We believe that the mutant mice studied represent a fascinating model to address the problem of hair loss. We demonstrated alterations in the morphogenesis of embryonic hair follicle in *we/we wal/wal* double homozygous mice developing alopecia postnatally. We suppose that incorrect morphogenesis of hair follicles during embryogenesis is closely related to alopecia in the adult life. Unveiling the mechanisms involved in altered embryogenesis may elucidate the pathogenesis of alopecia.

## 1. Introduction

Alopecia is a common clinical condition in dermatological practice. It is manifested in gradual or massive loss of hair [1]. There are a number of murine models of alopecia including spontaneous mutations [2–4]. In particular, symptoms closely resembling human alopecia were found in double homozygous mice* we/we wal/wal*, which become almost totally bald with small hairy patches by postnatal day 21 [5, 6].

**Figure 1:**
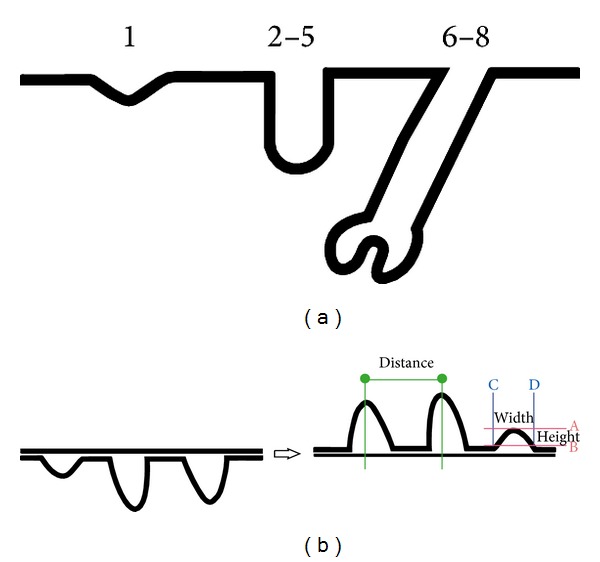
(a) Schematic outline of the epithelium invagination and downgrowth during hair follicle morphogenesis. (b) Schematic representation of the epidermis whole-mount analysis.

Hair follicle morphogenesis is a complex process involving temporal series of epitheliomesenchymal cross-talk [7–9]. Eight stages of hair morphogenesis are distinguished [10]. It is initiated at approximately E14.5. First morphological signs include the polarization of keratinocytes in epidermal placodes and the formation of dermal condensates beneath. Then placodes undergo marked proliferation and downgrowth to produce first the hair germs and then hair pegs. Finally, epithelial cells surround the dermal condensate and, thus, form the hair bulb of the mature follicle (Figure 1(a)).

Molecular signals regulating cycling of hair follicles in postnatal skin are very close to those found in embryo development [11–15]. So, we suggested that hair loss in mutant mice may be connected with some alterations in hair follicle development in embryogenesis.

Embryonic day 18.5 is a widely accepted term to study the structure of mouse skin in the course of follicle development. Four types of hair follicles found in the mouse fur (guard, awl, auchene, and zig-zag) are formed in the epidermis by this time [16]. The first thing to address in order to evaluate the process of folliculogenesis is the number and size of hair follicles. To this end, we used light and fluorescent microscopy combined with immunohistochemistry to study the patterning of hair follicles in embryonic epidermis of mutant mice in comparison with C57Bl6 counterpart. We counted the number of follicles on the whole-mounts under fluorescent microscope after staining with anti-P-cadherin antibodies. Additionally, we took the advantage of special options in the Keyence VHX-1000 digital microscope to obtain 3D images to measure the width and height of hair follicles at different stages of morphogenesis and to measure the distance between neighboring follicles. Our observations demonstrate alterations in the morphogenesis of embryonic hair follicle in* we/we wal/wal* double homozygous mice developing alopecia postnatally.

## 2. Materials and Methods

All experiments were performed in compliance with the guidelines for the care and use of laboratory animals established at the Institute of Developmental Biology. We used wild type mouse strain C57Bl6 and double homozygote mutant mice* we/we wal/wal*. The mutant mice were generated by the crossing of* we/we wal/wal* and C57BL/6 background mouse strain and kindly provided by Koniukhov et al. [5] and Nesterovaet al. [6]. Pregnant females were euthanized by cervical dislocation. Skin fragments from the dorsal side of E18.5 mouse embryos were used for the whole-mount specimens. Whole-mount epidermis specimens were prepared as described before [17] with modifications. Briefly, skin pieces were washed with Hank's balanced salt solution (HBSS) containing 80 *μ*g/mL gentamicin and incubated in 0.05% Dispase in DMEM for 12 h at +4°C. After that the epidermis was gently separated from the underlying dermis and transferred to PBS.

For light microscopy, whole-mounts were fixed in 2% glutaraldehyde in PBS for 30 min at room temperature, washed twice for 5 min with PBS, postfixed in 1% osmium tetroxide solution for 45 min at room temperature, washed two times for 5 min with PBS, dehydrated in a graded ethanol series, and air-dried. Dried epidermis was mounted to the microscope stand with the basal layer up (Figure 1(b)). Hair follicle patterning and morphology were analyzed using a Keyence VHX-1000 microscope. Average distance between follicles was calculated from 33 measurements in each group (wild-type mice and mutants). Differences were judged as significant if the *P* value was <0.05, as determined by the Student's *t*-test for independent samples. The width and the height were measured in 25 follicles for each group of animals. The difference in the average width between the control group and mutant one was judged as significant if the *P* value was <0.05, as determined by the Mann-Whitney *U* test.

For immunofluorescent analysis, total Е18.5 epidermis specimens were fixed in 4% paraformaldehyde solution in PBS for 30 min and then washed several times with PBS. Finally, whole-mount samples of epidermis were permeabilized by incubation in 0.1% Triton X-100 (AppliChem) and 0.1% Tween 20 (AppliChem) in PBS for 30 min and used for immunohistochemistry. The primary anti-P-cadherin goat polyclonal antibodies (AF 761, R&D systems) were diluted 1 : 80 in PBS containing 0.1% Triton X-100 and 5% BSA and incubated overnight at +4°C. The epidermis was then washed several times with PBS and incubated with secondary anti-goat antibodies conjugated with Alexa 488 (Molecular Probes, diluted 1 : 1000) in PBS for 1 h at room temperature. After rinsing with PBS, specimens were mounted in TDE medium (Abberior). Hair follicle morphogenesis was analyzed using an Olympus IX51 fluorescent microscope. Hair follicles were counted on 12 images (5.4 × 10^5^ *μ*m^2^) obtained from each group at a magnification of ×100. Total of 1458 follicles at different stages of morphogenesis in the wild-type group and 1001 in the mutant group were clearly identified using Image J. Differences were judged as significant if the *P* value was <0.05, as determined by the Mann-Whitney *U* test.

## 3. Results

**Figure 2:**
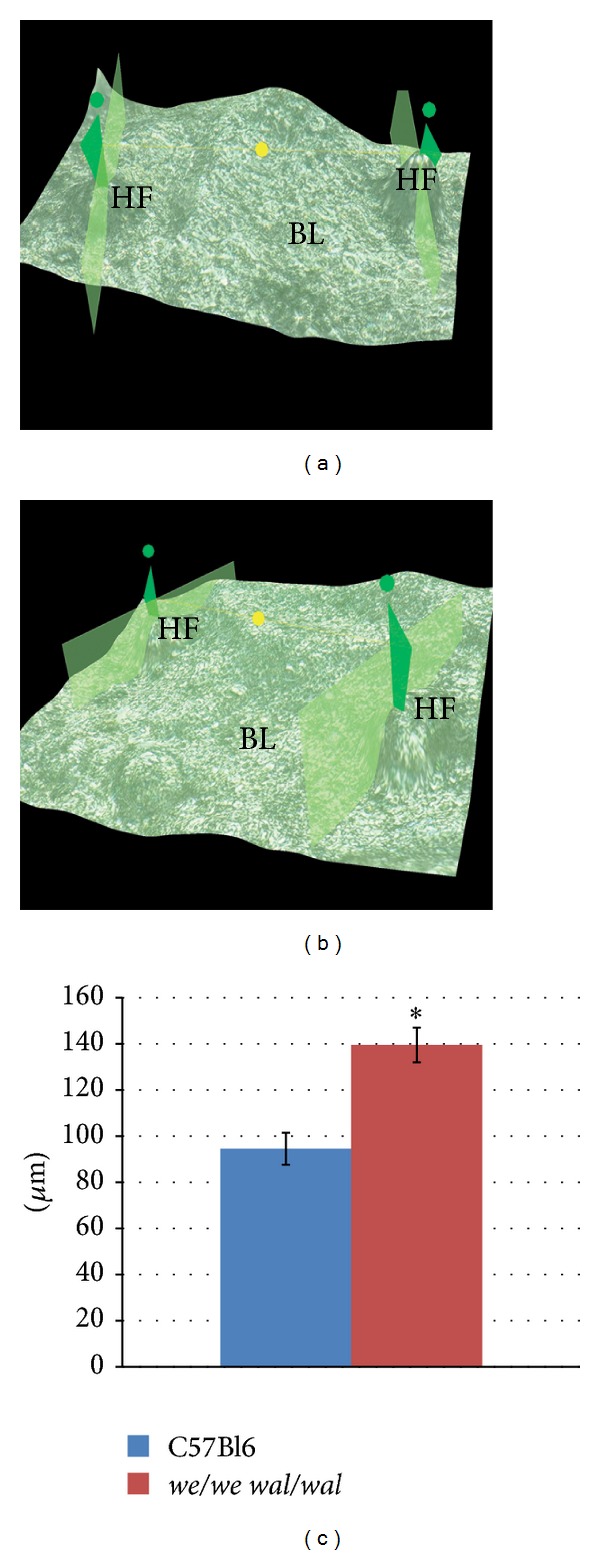
Distance measurements on 3D images of E18.5 epidermis whole-mount (Keyence VHS-1000). (a) Control C57Bl6 epidermis; ×1500. (b) Mutant epidermis; ×1500. (c) Graphical plot demonstrating average distance between neighboring follicles. Results are presented as mean ± SEM with the level of significance *P* < 0.05. The distance between two neighboring follicles of any size is significantly increased in mutants in comparison with C57Bl6 embryos, as indicated by the asterisk. Green point: the tip of the follicle, green box: cross section of the hair follicle; the distance between two green points is the distance between follicles, *μ*m. Hair follicle: HF. Basal layer: BL.

Hair follicle patterning in E18.5 epidermis of* we/we wal/wal* mice was compared to that of C57Bl6 embryos. To evaluate the density of hair follicles, we measured the distance between neighboring hair follicles and the height and width of follicles. Using Keyence VHX-1000 digital microscope and the software attached, we were able to determine the distance between the tips of individual follicles (corresponding to the lowest part of the growing follicle in the native epidermis). The follicles looked like elevating outgrowths when the epidermis was viewed upside down (Figure 1(b)). Measurements showed that the distance between two neighboring follicles of any size was significantly increased in mutants as compared to C57Bl6 embryos (139.51 ± 7.51 versus 94.55 ± 6.94 *μ*m) (Figure 2). Therefore, hair follicle primordia were more sparsely distributed in mutant embryonic epidermis. This may indicate reduced number of hair follicles in the mutant epidermis.

**Figure 3:**
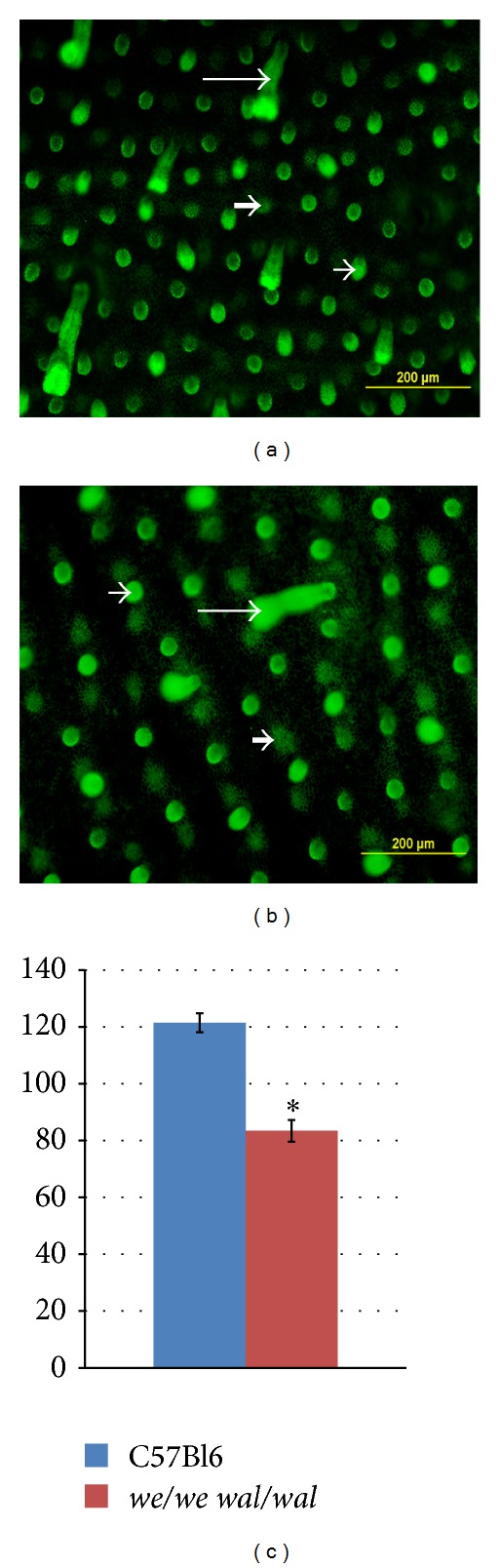
General view of E18.5 epidermis whole-mount. ((a), (b)) Fluorescent microscopy, green—P-cadherin staining. (a) Control C57Bl6 epidermis. (b) Mutant epidermis. (c) Average hair follicles score counted on the standard areas of fluorescent preparations. Results are presented as mean ± SEM with the level of significance *P* < 0.05. The number of follicles in mutant embryos is significantly decreased in comparison with C57Bl6 mice, as indicated by the asterisk. Early stage is shown by the thick arrow, medium stage is shown by the short arrow, and advanced stage is shown by the long arrow.

To look over the panoramic view of the epidermis, we utilized fluorescent microscopy with immunostaining of the whole-mount epidermal sheets for P-cadherin. This enabled us to visualize tightly packed P-cadherin-positive cells designating the presence of follicles (Figures 3(a) and 3(b)). Long follicles at advanced stages were seen as cylinders adhered to the interfollicular epidermis. Earlier stages of hair follicles looked like bright green spots. Medium stages appeared like green columns (Figures 3(a) and 3(b)). Therefore, in control specimens, the size of follicle primordia varied depending on the stage of follicle development. And all types of mouse hair were present at E18.5 in the epidermis as it had passed through several waves of follicle induction. In mutants, the number of follicles at different stages of development counted in one standard field was nearly 1.5-fold lower than in C57Bl6 embryos (83 ± 3.82 follicles in mutants versus 121 ± 3.36 in control embryos) (Figure 3(c)). Additionally, we measured the distance between the neighboring follicles on fluorescent images and confirmed the increased distance between mutant primordial follicles (data not shown). We also noticed that the early stages of follicles seemed to be bigger in mutant embryos in comparison with normal counterparts.

**Figure 4:**
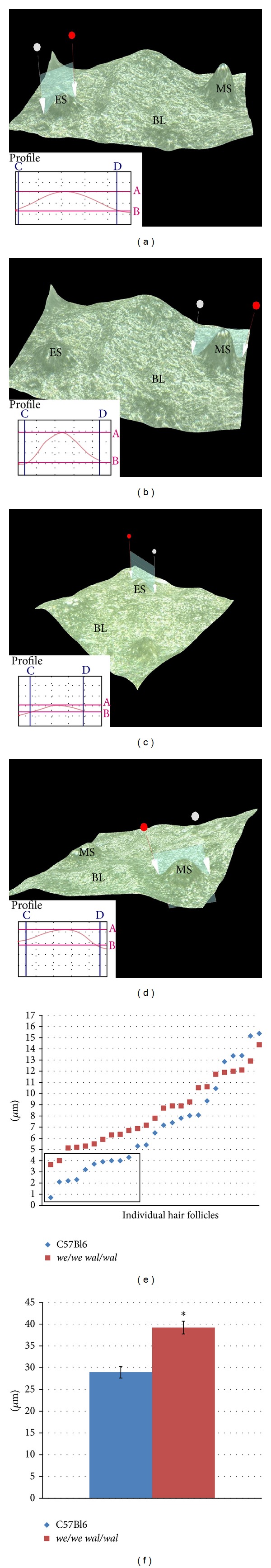
3D image of E18.5 epidermis whole-mount, follicle measurements (Keyence VHX-1000). ((a), (b)) Control C57Bl6 epidermis. ((c), (d)) Mutant epidermis. ((a), (c)) Early stage follicles. ((b), (d)) Medium stage follicles. (e) Individual height values of primordial follicles, early stages (up to 4 *μ*m) are outlined with the box. Height values of the individual hair follicles were sequentially distributed in increasing order. (f) Average width at the base of hair follicles. Results are presented as mean ± SEM with the level of significance *P* < 0.05. Mutant follicles are significantly wider than control ones, as indicated by the asterisk. ((a), (b), (c), (d)) Earlier stage: ES. Basal layer: BL. Medium stage: MS. The distance between pink lines A and B corresponds to the height and the distance between blue lines C and D corresponds to the width. White square is the cross section of the hair follicle. Red point is the beginning of the follicle section; white point indicates the completion of the section.

Analysis of light microscopic 3D images using the attached software allowed measurements of the height of follicles and their width at the points where they enter the interfollicular epidermis (at the “basis” of follicles as it was seen on images). Very early stages of developing follicles (we supposed that they correspond to the placode and the germ or to stages 1 and 2, resp., according to the classification by Paus et al. [10]) looked like small outgrowths not more than 4 *μ*m high (Figures 4(a) and 4(c)). Later stages were up to 16 *μ*m high (Figures 4(b) and 4(d)).  Figure 4(e) represents the histogram of the distribution of measured follicles according to their height. It is a continuous line of values from the smallest primordia to the biggest ones. As it was mentioned, the smallest values correspond to initial stages of hair follicle morphogenesis. The data obtained showed that the smallest values of the height were almost undetectable in mutant epidermis which seemed to be devoid of such follicles (Figure 4(e)). On average, mutant primordial follicles were wider than control ones (Figure 4(f)). As it could be predicted from the height measurements, the smallest values of the width corresponding to early stages of hair follicle development were almost undetectable in mutant epidermis in contrast to the control specimens where the follicles with the smallest width of nearly 27 *μ*m and less corresponded to those with the height of 4 *μ*m and less, thus affecting the average value (Figure 4(f)). It should be noted that we were unable to visualize single maximally developed follicles, which were clearly seen on fluorescent images and seemed to be around 200 *μ*m high. This is accounted for by specific preparation stages before light microscopy including drying of specimens, which leads to adhesion of long follicles to interfollicular epidermis. Thus, their morphometric parameters could not be measured properly.

## 4. Discussion

Hair follicle is a fascinating model for fundamental developmental biology because it is an organ that goes through regeneration in the adult under physiological condition. On the other hand, there is a growing incidence of hair follicle diseases including alopecia in humans [18–21].

Mechanisms of hair induction in embryogenesis and hair cycling in adult animals are closely related. Thus, searching inside embryo hair follicle morphogenesis may complement our knowledge about alopecia pathogenesis. There are numerous studies of mutant and transgenic mice with hair follicle impairments. However, most of them are focused on the postnatal period evaluating morphology and cycling of hair follicles [22–26].

Double homozygous* we/we wal/wal* mice are born hairy but demonstrate progressing alopecia and impaired hair cycling with prolonged catagen stage [6]. Using whole-mount epidermis of E18.5 embryos, we show here that hair follicle defects can be revealed during embryonic morphogenesis in these mutants.

It was demonstrated earlier that the whole-mount labeling of epidermal sheets can be used to visualize expression of stem cell markers [27], to trace label-retaining cells [17] and sebocytes [28], and so forth. We used this approach to study hair follicle patterning in embryo epidermis of mutant* we/we wal/wal* mice in comparison with C57Bl6 counterparts. We explored P-cadherin expression to identify hair follicle primordia. E- and P-cadherins are classical cadherins, which are a major component of adherens junction and participate in many biological processes such as cell recognition, cell signaling, embryonic cell migration, and tumor development [29]. Upon formation of the hair follicle placode, the expression of E-cadherin is markedly downregulated, while P-cadherin is simultaneously upregulated [30, 31].

Hair follicle primordia were of different sizes in E18.5 epidermis. This implies that primordia of different types of hair follicles were present at this instant. Actually, it is well known that discrete waves of hair follicles are observed in embryo epidermis beginning at approximately E14.5 and ending near birth [32]. The sequence of hair follicles emergence looks as follows: Е14.5: guard; E16.5: awl/auchene; and E18.5-P1: zig-zag [16].

We found that the number of follicles was decreased in mutant embryonic epidermis. This may indicate that hair follicle morphogenesis is retarded in mutant mice as young pups are hairy. In a sense, we can speculate that first signs of alopecia are manifested during embryonic development.

Placodes are the most difficult structures to identify in the whole-mount epidermis, both by fluorescent and light microscopy. All basal keratinocytes express P-cadherin to some extent but placodes show the highest level of expression [31]. However, sometimes it is rather difficult to fix the placode in the whole-mount preparation as the number of cells comprising the placode may vary. Later-staged follicles were easily observed as bright green “hills.” If you deal with severe morphogenesis alterations, you will readily see them using whole-mount fluorescent analysis. In our case, we noticed immediately the reduced number of follicles and increased diameter of nascent follicles. To study parameters of hair follicles in detail, we used light microscopy with additional options. We found very few placodes in mutant epidermis as compared to the normal one. Noteworthy, we observed some structures resembling the placodes on the immunofluorescent images. They looked like bright patches (indicating tightly packed cells) with irregular shape (indicating defect arrangement of cells inside the placode). Therefore, mutant placodes failed to provide adequate volume to be captured by light microscope. It may be supposed that such defect placodes were further unable to form the follicles.

Increased width of the hair follicles in mutant embryos may be related to improper inductive signal from the mesenchyme as the size of the hair follicle is determined by the size of the dermal papilla [33]. Another point is that normal keratinocytes at the base of nascent follicles exhibit dramatic changes in cell shape, cytoskeletal organization, adherens junction formation, intercellular adhesion, and epithelial polarity [34]. Increased diameter and irregular shape of the nascent follicles may indicate some defects in this process.

## 5. Conclusions

The mutant mice studied represent an interesting model to address the problem of alopecia. We discovered serious impairments of hair follicle patterning in the developing epidermis of mutant* we/we wal/wal* mice. We propound that incorrect morphogenesis of hair follicles during embryogenesis is closely related to alopecia in the adult mice. Unveiling the mechanisms involved in altered embryogenesis may elucidate the pathogenesis of alopecia. Our approach may be useful for quick evaluation of the pattern and degree of hair follicle development in mutant mice with “skin phenotype.”
